# Component-Specific Advantages in Visual Attention Across Experience Groups: A UFOV Study

**DOI:** 10.3390/bs16040513

**Published:** 2026-03-29

**Authors:** Siyu Guo, Ziyao Liu, Lu Yin, Zhao Li, Yingzhi Lu

**Affiliations:** 1 School of Psychology, Shanghai University of Sport, Shanghai 200438, China; 2321974009@sus.edu.cn (S.G.); 2421974010@sus.edu.cn (Z.L.); 2 School of Physical Education and Health, Shanghai University of International Business and Economics, Shanghai 201620, China; 8020@suibe.edu.cn; 3 Department of Physical Education, Zhejiang Gongshang University, Hangzhou 310018, China; lz2024026@mail.zjgsu.edu.cn; 4 Key Laboratory of Motor Cognitive Assessment and Regulation, Shanghai University of Sport, Shanghai 200438, China

**Keywords:** visual attention, Useful Field of View (UFOV), attentional control, selective attention, divided attention, experience-related differences

## Abstract

Visual attention involves the efficient allocation of processing resources across space and under conditions of visual competition. This study examined whether experience-related advantages in visual attention are expressed uniformly or selectively across attentional components. Using a modified Useful Field of View (UFOV) paradigm, four groups with distinct experiential backgrounds were compared: table tennis players (TTPs), action video game players (AVGPs), aerobic gymnastics athletes (AGAs), and non-trained college students (NCSs). Subtest 1 assessed central identification under relatively low attentional control demands. No significant group differences were observed, indicating comparable basic central identification performance across groups. Subtests 2 and 3 assessed divided attention and selective attention under interference, respectively. In Subtest 2, all experienced groups outperformed NCSs, with no differences among TTPs, AVGPs, and AGAs. In Subtest 3 under high visual competition, performance diverged; TTPs and AVGPs outperformed both AGAs and NCSs, whereas AGAs did not differ from controls. These findings indicate that experience-related advantages in visual attention are component-specific rather than global, and become most evident when tasks place stronger demands on attentional control under interference. The advantage pattern shown by TTPs under higher attentional control demands was more compatible with visually demanding experience than with physical training alone. No significant interactions with eccentricity were observed, suggesting consistent group differences across peripheral distances.

## 1. Introduction

Visual attention enables individuals to extract task-relevant information from complex environments under time and capacity constraints (Evans et al., 2011), which is central to performance across a wide range of everyday and professional competitive contexts. For example, during driving, attention is primarily anchored on the roadway ahead, while peripheral monitoring of adjacent vehicles and pedestrians remains essential. In fast-paced ball sports, athletes must maintain continuous observation of an opponent’s actions while simultaneously tracking a rapidly moving object. Across these situations, successful performance depends on coordinating central processing with peripheral monitoring and exerting control over irrelevant visual interference. Accordingly, visual attention is fundamentally a problem of attentional resource allocation and control, rather than a simple measure of visual acuity or sensory capacity (Posner & Boies, 1971; Wolfe & Horowitz, 2004). At the same time, visual attention is not fixed, but plastic, and can be shaped by experience and training (Edwards et al., 2005; Mandolesi et al., 2018). Against this background, an important question is whether different experiential backgrounds are associated with different forms of attentional advantage.

Among such backgrounds, action video game experience provides one of the clearest examples of experience-related differences in visual attention. A large body of research has shown that action video game players often demonstrate superior efficiency under conditions of high information density, multiple competing objects, and visual interference, including faster target selection, broader visuospatial attentional distribution, and more effective suppression of irrelevant information in complex scenes (Bavelier & Green, 2004; Green & Bavelier, 2006, 2007; Oei & Patterson, 2015). These advantages are commonly interpreted as being related to the repeated demands for rapid selection, flexible attentional shifting, and peripheral monitoring in action games (Bejjanki et al., 2014; Green et al., 2010). Importantly, similarly demanding visual environments are not unique to action video games. Open-skill sports, especially fast interactive ball sports such as table tennis, also require performers to integrate information from multiple sources in continuously changing situations, extract critical cues, rapidly judge the dynamic changes of opponents and objects, and generate timely and precise responses (Lees, 2003; Presta et al., 2021). Research on perceptual–cognitive expertise in sport further suggests that expert advantages are not limited to general physical fitness, but are also reflected in sport-relevant operations such as visual search, cue utilization, anticipation, and selective attention (Del Campo et al., 2011; Logan et al., 2023; Mann et al., 2007; Scharfen & Memmert, 2019). Thus, from the perspective of experiential background, action video game players and open-skill athletes may both be repeatedly exposed to environments characterized by high information density, strong time pressure, and high visual competition, albeit in different activity forms.

Although existing studies generally suggest that both action video game experience and sport experience may be associated with better visual attention, the form of such advantages remains insufficiently specified. The literature has often described experience-related differences as reflecting a relatively general attentional enhancement, but has less often tested whether these advantages are expressed uniformly across all components of visual attention. In fact, because visual attention itself consists of multiple functionally distinct components, experience-related advantages may not generalize across components in the same way. Early research on transfer and practice specificity has suggested that transfer effects usually depend on the overlap in processing demands between prior experience and the measured task, rather than automatically extending to all cognitive operations (Franks et al., 2000; Morris et al., 1977; Proteau et al., 1992; Woltz et al., 2000). This issue can also be understood more clearly from theories of attentional control. The distinction between goal-directed (top–down) attention and stimulus-driven attention suggests that tasks differ in the extent to which they require active attentional allocation, selection, and inhibition (Corbetta & Shulman, 2002; Sawaki & Katayama, 2008). Executive control theories further suggest that individual differences are more likely to emerge when successful task performance depends on maintaining goals, biasing processing toward task-relevant information, and resolving competition between stimuli (Desimone, 1998). Accordingly, one key but still insufficiently answered question in research on experience and visual attention is whether experience-related advantages reflect a component-general enhancement across attentional components, or instead a component-specific pattern that matches the attentional operations repeatedly trained by prior experience.

Answering this question requires more than relying on the heterogeneous task paradigms used in previous studies. Many studies on action video game experience or sports experience involve visual attention, but they differ considerably in stimulus format, task demands, and outcome measures. As a result, it is difficult to directly compare which specific components of visual attention are most likely to be influenced by different experience backgrounds (Manci et al., 2025). For this reason, the present study adopted the Useful Field of View (UFOV) task as a unified measurement framework. A major advantage of the UFOV is that it allows for multiple functionally related but demand-distinct attentional components to be assessed under the same threshold-based logic. In Subtest 1, participants are mainly required to rapidly identify a central target. This Subtest can be used to assess central processing efficiency under relatively low attentional control demands. In Subtest 2, participants must localize a peripheral target while also completing central identification. This Subtest therefore places greater emphasis on goal-directed attentional allocation between central and peripheral regions. In Subtest 3, distractor information is added around the peripheral target. This further assesses selective attention and inhibitory control under visual interference (Ball & Owsley, 1993; Edwards et al., 2005; Sanders, 1970; Wood & Owsley, 2014). In addition, previous work has shown that peripheral target eccentricity is related to peripheral localization and UFOV performance, although findings have been mixed regarding whether eccentricity further moderates UFOV patterns in tasks involving peripheral localization (Gaspar et al., 2016; Owsley et al., 1995; Schieber & Gilland, 2005). To further examine whether experience-related differences would be modulated by peripheral processing demands, the present study included two eccentricity levels (10° and 16°) in Subtests 2 and 3 and compared performance patterns across these peripheral conditions. By comparing relative performance patterns across these Subtests within the same paradigm, UFOV provides a more direct means of examining whether experiential advantages are global or component-specific. At the same time, the standard UFOV protocol was originally developed mainly for older adults and clinical populations (Ball & Owsley, 1993; Na et al., 2024; Sekuler et al., 2000), and may be insufficiently sensitive for young high-performing individuals because of relatively simple stimuli and task demands (Wood & Owsley, 2014). Therefore, the present study retained the core measurement logic of UFOV while increasing stimulus richness and interference strength to improve sensitivity for young participants and high-performing groups.

However, even if some experiential backgrounds show similar advantages in specific attentional components, the experiential interpretation of those patterns may still differ. In the case of sport experience, the superior performance often observed in open-skill athletes on attentional and perceptual–cognitive tasks may be associated either with long-term exposure to environments characterized by high information density, strong time pressure, and continuous dynamic updating, or with long-term systematic training, greater physical load, and better fitness status (Lees, 2003; Presta et al., 2021; Zhao et al., 2023). Because physical activity and aerobic exercise have also been linked to attentional allocation and cognitive functioning more generally (Logan et al., 2023; Mandolesi et al., 2018; Scharfen & Memmert, 2019), comparisons between a single open-skill athlete group and non-athlete controls are usually insufficient to distinguish whether the observed pattern is more consistent with a high visual–attentional demand background or with long-term physical training-related differences (Scharfen & Memmert, 2019). On this basis, the present study adopted a four-group design: table tennis players (TTPs), who combine high visual–attentional demands with long-term systematic training; action video game players (AVGPs), who are exposed to high visual–attentional demands without corresponding sport-specific physical training; aerobic gymnastics athletes (AGAs), who have long-term systematic training and high physical load but whose experience background does not emphasize the same type of dynamic visual competition as table tennis or action video games; and non-trained college students (NCSs), who served as a low-relevance comparison group. The present study compared the relative performance patterns of the four groups across UFOV components. It also examined whether these patterns varied across different eccentricity conditions. This design allowed us to test whether the attentional profile of open-skill athletes was more similar to that of participants with high visual–attentional demands or to that of participants with long-term physical training.

Based on the above considerations, the present study used a modified UFOV framework to compare the performance patterns of TTPs, AVGPs, AGAs, and NCSs across different attentional components. The primary aim was to examine whether experience-related differences would appear as component-general advantages across UFOV subtests or as selective component-specific patterns, and whether the attentional profile of open-skill athletes would be more consistent with experience in high visual–attentional demand contexts or with long-term physical training. Accordingly, three hypotheses were proposed.

**Hypothesis** **1.** *If experience-related advantages are component-specific rather than component-general, group differences should not emerge uniformly across all UFOV subtests, but should be more evident in subtests that place greater demands on specific attentional operations.*

**Hypothesis** **2.** *Because Subtests 2 and 3 place greater demands than Subtest 1 on goal-directed attentional allocation between central and peripheral targets, rapid target selection, and distractor control, we expected experience-related differences to be more evident in these subtests, particularly in Subtest 3.*

**Hypothesis** **3.** *If group differences emerge in Subtests 2 and 3, the relative pattern of similarity between TTPs and the other experienced groups may help distinguish between two experiential interpretations. If the attentional profile of open-skill athletes is more compatible with experience in high visual–attentional demand contexts, TTPs should show a pattern more similar to AVGPs than to AGAs. In contrast, if this profile is more consistent with long-term physical training, TTPs should show a pattern more similar to AGAs than to AVGPs.*

## 2. Methods

### 2.1. Participants

We initially recruited 108 participants. Prior to analysis, four participants were excluded: one table tennis player (TTP) who did not complete all experimental blocks, and three aerobic gymnastics athletes (AGAs) whose UFOV threshold performance fell more than 3 standard deviations from the sample mean. The final analytic sample therefore consisted of 104 participants, including 24 table tennis players (TTPs), 25 action video game players (AVGPs), 28 aerobic gymnastics athletes (AGAs), and 27 non-trained college students (NCSs). The sample size was estimated using G*Power 3.1.9.7. for the primary hypothesis. A medium effect size (f = 0.25) was specified for the F-test, with statistical power set at 0.95 and alpha at 0.05. The minimum required total sample size was 72. The final analytic sample of 104 participants therefore exceeded the planned requirement and provided adequate statistical power for all primary analyses.

All athletes (TTPs and AGAs) were at least national first-level athletes according to the Chinese sport classification standards and had engaged in systematic training for at least eight years. The AVGPs met the action video game player (AVGP) criteria based on the Bavelier Lab Video Game Questionnaire (VGQ) obtained from the Brain and Learning Lab (University of Geneva). In line with commonly used thresholds, AVGPs were defined as individuals who currently played ≥5 h/week of action video games and ≤3 h/week of non-action games (Green et al., 2017). The NCSs reported minimal video game experience and no formal training experience in table tennis or aerobic gymnastics. In addition, during recruitment, each participant underwent verbal screening to minimize overlap in experience backgrounds across groups. NCS participants were screened to confirm that they had no regular participation in other ball sports, shooting-related activities, or other backgrounds involving frequent visual attention demands, and no regular spectating exposure to such activities. Athlete participants were screened to confirm that they had no regular participation in esports or action video games, whereas AVGP participants were screened to confirm that they had no formal training in sports relevant to the present study. Participants reporting such conflicting backgrounds were not included. They were right-handed, healthy young adults with normal or corrected-to-normal vision and normal color vision. To minimize potential confounds associated with expertise, only individuals with less than one year of driving experience were included (Crundall, 2016). Detailed demographic characteristics, including age, gender, training years, weekly training or gaming hours, are summarized in Table 1.

All were informed of the specific requirements of the experiment and signed an in-formed consent form before participating. All procedures adhered to the Declaration of Helsinki. The experiment was approved by the Ethics Committee of the Shanghai University of Sport (Approval No. 102772025RT111).

### 2.2. Apparatus and Stimuli

Stimuli were presented on a 1920 × 1080-pixel LCD monitor (screen width 47.5 cm, height 26.5 cm). Participants were seated at a viewing distance of 42 cm, with their head position stabilized by a chin rest. These parameters were used to convert visual angles (in degrees) into pixel coordinates for stimulus presentation.

The experimental paradigm was adapted from standard Useful Field of View (UFOV) tasks used to assess visual spatial attention, but was modified in terms of stimulus set and eccentricities. The background of the display was black throughout all Subtests. Across all Subtests, the central identification stimuli consisted of three green vehicle icons (a truck, a tractor, and a car). In Subtests 2 and 3, a peripheral target stimulus, a green train front was presented at one of several predefined peripheral locations. In Subtest 3, an additional distractor stimulus was introduced: a green trolley bus, which appeared only as a distractor and was never used as a central stimulus or as the peripheral target.

**Figure 1:**
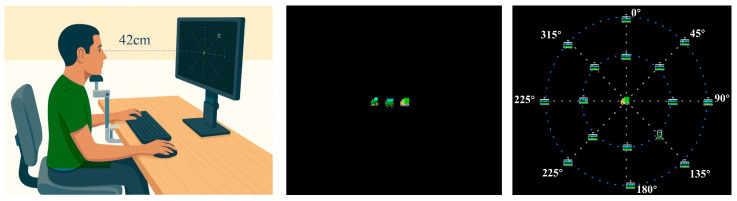
Experimental setup. The participant viewed the screen from a fixed distance using a chin rest. The screen displays the spatial arrangement of the stimuli used in the UFOV Subtest 3. The colored icons represent the stimulus types used in the task, and the dashed concentric rings are shown for illustration purposes only and were not displayed during the actual task.

Peripheral stimuli were presented at 16 possible locations arranged on two concentric rings centered on the fixation point. Each ring contained eight positions equally spaced at 45° intervals (0°, 45°, 90°, 135°, 180°, 225°, 270°, and 315°). The inner ring was located at an eccentricity of 10°, and the outer ring at 16° of visual angle from the fixation. The spatial positions of the peripheral stimuli were defined in degrees of visual angle and converted to screen coordinates based on the viewing distance and screen geometry using trigonometric conversion. All stimulus icons subtended approximately 1° × 1° of visual angle. Figure 1 illustrates the experimental apparatus and the visual stimuli.

Stimulus presentation and response collection were controlled using MATLAB R2021a (MathWorks, Natick, MA, USA) and Psychtoolbox 3.0.18 extensions.

### 2.3. Tasks and Procedure

Each participant completed three UFOV Subtests in fixed order with increasing attentional demands: (1) central processing speed, (2) divided attention, and (3) selective attention with distractors. This fixed sequence was used to preserve the progressive structure of the modified UFOV paradigm and to maintain procedural consistency across participants. All tasks followed an adaptive staircase procedure (3-down–1-up) to estimate individual thresholds in stimulus display duration. Between Subtests, participants were given a rest period of approximately five minutes to reduce fatigue and attentional lapses and to minimize immediate carry-over between subtasks. A short practice phase preceded each Subtest to familiarize participants with the stimuli and response requirements.

#### 2.3.1. General Trial Structure

Across all Subtests, participants were required to perform a central identification task, indicating which vehicle stimulus was presented at fixation using labeled keyboard keys. In Subtests 2 and 3, participants additionally performed a peripheral localization task, reporting the perceived location of the peripheral target by clicking on the display with a mouse. On each trial, a central fixation point was presented to standardize initial gaze at screen center prior to stimulus onset, followed by a Subtest-specific stimulus display. The stimulus display was presented for a brief duration that was initially set at a predefined value and subsequently adjusted by an adaptive staircase procedure, after which the stimuli disappeared. The adaptive staircase followed a 3-down–1-up rule (i.e., the display duration decreased after three consecutive correct responses and increased after any incorrect response). Step size was defined in units of screen refresh frames: the initial step size was 2 frames and was reduced to 1 frame after the third reversal. A reversal was registered whenever the adjustment direction changed (i.e., from increasing to decreasing or vice versa) across successive non-zero updates. On each update, the duration was adjusted by adding or subtracting the step size multiplied by the inter-frame interval (IFI), and durations were constrained to be no shorter than 1 frame. For Subtests 2 and 3, two independent staircases were maintained for the inner ring (10°) and the outer ring (16°), and only the staircase corresponding to the presented eccentricity was updated on a given trial. A trial was considered correct only when all required responses were accurate (i.e., correct central identification in all Subtests and correct peripheral localization in Subtests 2 and 3). The staircase terminated when the predefined stopping criterion was reached (i.e., 8 reversals or 10 consecutive trials at the minimum duration), yielding threshold estimates. For each staircase, the threshold was defined as the mean display duration across the final five trials prior to termination.

#### 2.3.2. Procedure for Subtest 1: Central Processing Speed

**Figure 2:**
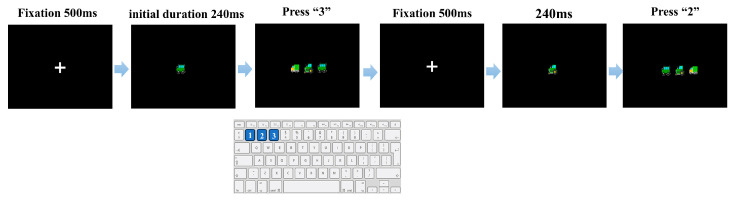
Experimental procedure of Subtest 1. The sequence depicts the presentation of the fixation cross (500 ms) followed by the target stimulus. The stimulus duration varies adaptively based on performance (initial duration = 240 ms). Participants respond to the subsequent choice array by pressing the key (“1”, “2”, or “3”) that spatially corresponds to the target’s identity. The bottom panel illustrates the schematic layout of the response keys used in the experiment.

In Subtest 1, each trial followed the sequence illustrated in Figure 2. A fixation cross was presented on a black background for 500 ms, followed by a brief central stimulus display in which one vehicle icon (truck, tractor, or car) appeared at the center of the screen. The initial display duration was set to 240 ms and was subsequently adjusted by an adaptive staircase procedure. Immediately after the stimulus display, a response screen appeared showing all three vehicle icons simultaneously. Participants indicated which icon had been shown in the preceding stimulus display by pressing one of three keys (1–3) with the left hand (ring/middle/index finger, respectively), corresponding to the left-to-right position of the icons on the response screen (Figure 2). The left-to-right arrangement of the three icons on the response screen was randomized on every trial to prevent fixed stimulus–response associations. No peripheral target or distractors were presented in this Subtest.

#### 2.3.3. Procedure for Subtest 2: Divided Attention (Central Identification and Peripheral Target)

**Figure 3:**
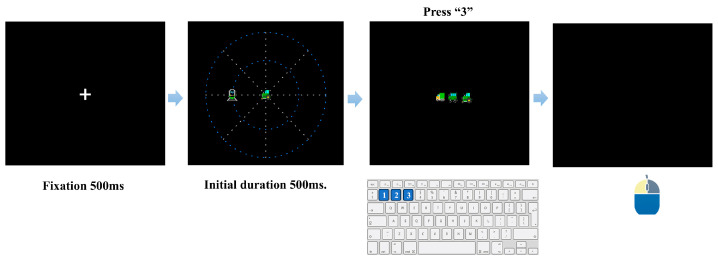
Experimental procedure of Subtest 2 (Divided Attention). The sequence initiates with a fixation cross (500 ms). Subsequently, a central target (for identification) and a peripheral target (for localization) are presented simultaneously. Participants first identify the central target via keypress (“1”, “2”, or “3”) and then indicate the peripheral target’s location using a mouse click. The dashed concentric rings are shown for illustration only and were not displayed during the experiment; the actual stimulus background was uniformly black.

Subtest 2 required participants to process a central stimulus and a peripheral target within the same trial. Each trial began with a fixation cross presented on a black background for 500 ms. A brief stimulus display then followed, in which one vehicle icon (truck, tractor, or car; randomly selected) appeared at fixation while a peripheral target icon (a locomotive) was presented simultaneously at one of 16 possible locations (Figure 3). These locations were defined by eight evenly spaced radial directions (45° apart) × two eccentricity rings (inner and outer, at approximately 10° and 16°, respectively; Figure 2). The central vehicle and peripheral target appeared and disappeared concurrently. The initial duration of this simultaneous stimulus display was set to 500 ms and was subsequently adjusted by independent adaptive staircases for inner- and outer-ring trials. After stimulus offset, a response screen displaying the three vehicle icons was presented, and participants first performed central vehicle identification using the same response procedure as in Subtest 1. The task then transitioned to peripheral localization on a target-absent black screen. Participants clicked the remembered location of the peripheral target without any on-screen grid or spatial markers (the 16-sector schematic in Figure 3 illustrates the possible target locations only and was not shown during the task). Both the keypress (central identification) and mouse-click (peripheral localization) stages had a 3000 ms response deadline; failure to respond within the time limit at either stage was scored as incorrect. Mouse localization accuracy was evaluated using a “sector + radius” criterion: clicks were considered angularly correct if they fell within ±22.5° of the target direction, and radially correct if they fell on the appropriate ring (inner-ring targets required clicks within a predefined boundary radius, whereas outer-ring targets required clicks beyond it; the boundary was set midway between 10° and 16°). A trial was scored as correct only when both central identification and peripheral localization responses were correct.

#### 2.3.4. Procedure for Subtest 3: Selective Attention with Distractors

**Figure 4:**
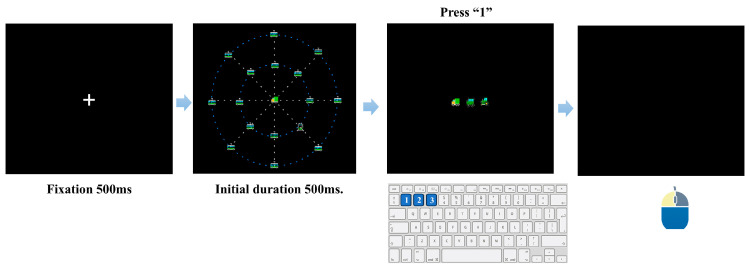
Experimental procedure of Subtest 3 (Selective Attention). The sequence follows the same structure as Subtest 2 but introduces visual clutter. Participants must identify the central target via keypress and subsequently locate the peripheral target amidst the distractors using a mouse click. The dashed concentric rings are shown for illustration only and were not displayed during the experiment; the actual stimulus background was uniformly black.

Subtest 3 was identical to Subtest 2 in trial sequence and response requirements (including a 500 ms fixation, central identification followed by peripheral localization, 3000 ms response deadlines for both stages, peripheral localization performed on a blank black screen without any spatial grid cues, the “sector + radius” criterion for mouse accuracy, and scoring a trial as correct only when both the central and peripheral responses were correct). The critical difference was the introduction of a peripheral distractor array during the simultaneous presentation of the central identification and peripheral localization stimuli, thereby imposing higher demands on selective attention and suppression of irrelevant distractors (Figure 4). Specifically, during the brief stimulus display, one vehicle icon (truck, tractor, or car; randomly selected) appeared at fixation, and the peripheral target (a locomotive) was presented at one of 16 possible locations defined by eight radial directions × two eccentricities (inner ring: 10°; outer ring: 16°). Unlike Subtest 2, the remaining 15 non-target peripheral locations were simultaneously filled with tram icons as distractors; these distractor icons were never used as either central targets or peripheral targets. The central stimulus, peripheral target, and distractors appeared and disappeared concurrently. The initial duration of this simultaneous stimulus display was set to 500 ms and was subsequently adjusted by independent adaptive staircases for inner- and outer-ring trials.

### 2.4. Statistical Analysis

Statistical analyses were conducted in accordance with the structural differences among the UFOV Subtests. Subtest 1 involved only central identification and yielded a single threshold estimate for each participant. It was therefore analyzed separately using a one-way ANOVA with Group (TTPs, AVGPs, AGAs, NCSs) as the between-subject factor.

By contrast, Subtests 2 and 3 both involved central identification combined with peripheral localization and included an additional within-subject manipulation of eccentricity (10° vs. 16°). As described in the Introduction, eccentricity was included as an additional within-subject factor to examine whether increased peripheral processing demands would alter the pattern of performance. Therefore, rather than collapsing across eccentricity, thresholds from Subtests 2 and 3 were analyzed using a 4 (Group: TTPs, AVGPs, AGAs, NCSs) × 2 (Subtest: 2, 3) × 2 (Eccentricity: 10°, 16°) mixed-design ANOVA, with Group as a between-subject factor and Subtest and Eccentricity as within-subject factors. When significant interactions or main effects involving Group were observed, follow-up simple-effects analyses and conditional pairwise comparisons were conducted. Pairwise comparisons were adjusted using the Holm correction for multiple testing. For these comparisons, mean differences (ΔM), 95% confidence intervals (CIs), adjusted *p* values, and Cohen’s d are reported. Effect sizes for ANOVA effects are reported as partial eta squared (η*_p_*^2^). Statistical significance was set at *p* < 0.05.

## 3. Results

### 3.1. Subtest 1: Central Processing Speed

**Figure 5:**
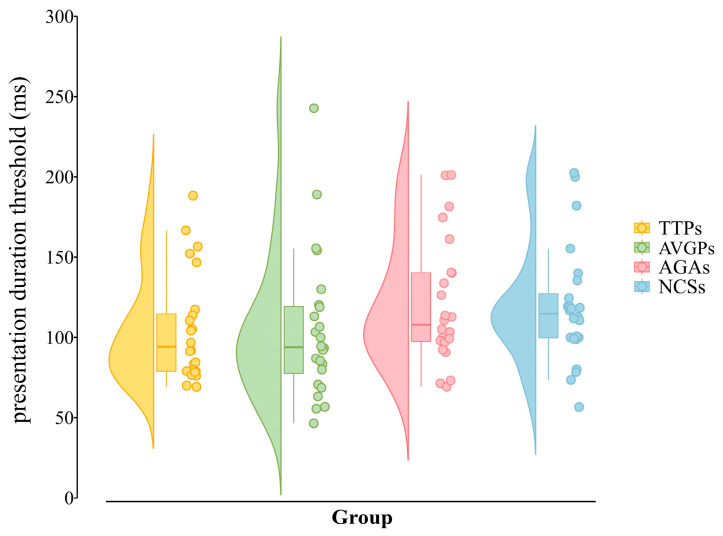
Presentation duration thresholds of the four groups in Subtest 1. Different colors represent the four groups (TTPs, AVGPs, AGAs, and NCSs). For each group, the violin plot shows the distribution of individual presentation duration thresholds, the embedded boxplot summarizes the central tendency and dispersion of the data, and the overlaid dots represent individual participants’ raw data. Higher threshold values indicate longer presentation durations required for correct central identification, and thus poorer performance.

A one-way between-subject ANOVA was conducted on the Subtest 1 presentation duration threshold with group (TTPs, AVGPs, AGAs, NCSs) as the between-subject factor. The main effect of group was not significant, *F*_(3, 100)_ = 1.32, *p* = 0.271, η*_p_*^2^ = 0.038, indicating comparable central identification speed across groups (TTPs: 105.12 ± 33.49 ms; AVGPs: 103.74 ± 44.38 ms; AGAs: 117.48 ± 37.47; NCSs: 121.11 ± 38.52). The distribution of presentation duration thresholds across groups is shown in Figure 5.

### 3.2. Subtests 2–3: Divided vs. Selective Attention

For Subtests 2 and 3, presentation duration thresholds were analyzed using a 4 (Group: TTPs, AVGPs, AGAs, NCSs) × 2 (Subtest: 2 vs. 3) × 2 (Eccentricity: inner 10° vs. outer 16°) mixed-design repeated-measures ANOVA. The analysis revealed significant main effects of Subtest, *F*_(1, 100)_ = 374.17, *p* = 1.428 × 10^−35^, η*_p_*^2^ = 0.789, and eccentricity, *F*_(1, 100)_ = 194.62, *p* = 3.353 × 10^−25^, η*_p_*^2^ = 0.661, indicating longer thresholds in Subtest 3 than Subtest 2 and longer thresholds for the outer ring than the inner ring. The main effect of group was also significant, *F*_(3, 100)_ = 14.33, *p* = 7.775 × 10^−8^, η*_p_*^2^ = 0.301.

Importantly, the Subtest × Group interaction was significant, *F*_(3, 100)_ = 3.50, *p* = 0.018, η*_p_*^2^ = 0.095, whereas the Eccentricity × Group interaction (*F*_(3, 100)_ = 0.18, *p* = 0.908, η_*p*_^2^ = 0.005), Subtest × Eccentricity interaction (*F*_(1, 100)_ = 1.54, *p* = 0.218, η_*p*_^2^ = 0.015), and the three-way interaction, *F*_(3, 100)_ = 1.56, *p* = 0.204, η_*p*_^2^ = 0.045, were not significant.

**Figure 6:**
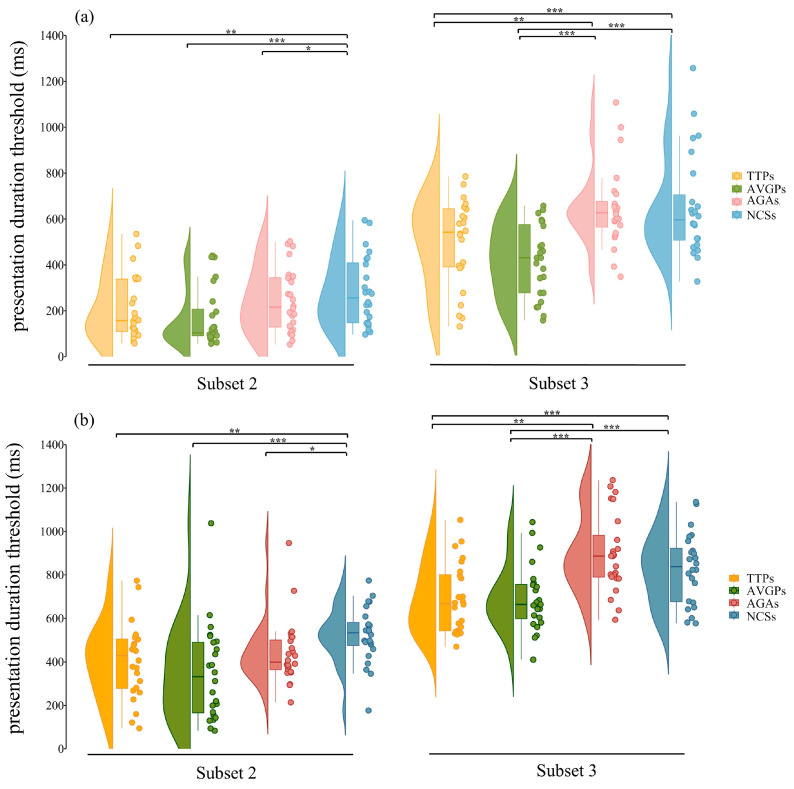
Presentation duration thresholds of the four groups across Divided Attention (Subtest 2) and Selective Attention (Subtest 3) conditions. The data are stratified by eccentricity: (**a**) Low-eccentricity (10°) and (**b**) High-eccentricity (16°). Within each eccentricity panel, the left side corresponds to Subtest 2 and the right side to Subtest 3. Different colors represent the four groups (TTPs, AVGPs, AGAs, and NCSs). For each group, the violin plot shows the distribution of individual thresholds, the embedded boxplot summarizes the central tendency and dispersion of the data, and the overlaid dots represent individual participants’ raw data. Higher threshold values indicate longer presentation durations required for correct identification/localization, and thus poorer performance. Horizontal brackets indicate significant between-group comparisons within each Subtest, and asterisks denote significance levels (* *p* < 0.05, ** *p* < 0.01, *** *p* < 0.001).

Given the significant Subtest × Group interaction, we examined the simple main effect of Group within each Subtest. Group differences were significant in both Subtest 2, *F*_(3, 100)_ = 8.72, *p* = 3.416 × 10^−5^, and Subtest 3, *F*_(3, 100)_ = 10.36, *p* = 5.363 × 10^−6^. Holm-adjusted conditional pairwise comparisons based on the estimated marginal means from the omnibus 4 × 2 × 2 mixed-design ANOVA, averaged across eccentricity, indicated that in Subtest 2, presentation duration thresholds were lower in TTPs than NCSs (ΔM = −106.93 ms, 95% CI [−192.26, −21.61], *p*__holm_ = 0.005, *d* = −0.64), in AVGPs than NCSs (ΔM = −156.10 ms, 95% CI [−240.51, −71.68], *p*__holm_ = 1.614 × 10^−5^, *d* = −0.93), and in AGAs than NCSs (ΔM = −91.91 ms, 95% CI [−173.94, −9.88], *p*__holm_ = 0.013, *d* = −0.55). No reliable differences were observed between TTPs and AVGPs (*p*__holm_ = 0.262), between TTPs and AGAs (*p*__holm_ = 0.634), or between AVGPs and AGAs (*p*__holm_ = 0.125). In Subtest 3, TTPs and AVGPs showed lower presentation duration thresholds than both AGAs (TTPs–AGAs: ΔM = −156.47 ms, 95% CI [−271.50, −41.44], *p*__holm_ = 0.002, *d* = −0.94; AVGPs–AGAs: ΔM = −186.52 ms, 95% CI [−300.30, −72.73], *p*__holm_ = 1.552 × 10^−4^, *d* = −1.11) and NCS (TTPs–NCSs: ΔM = −146.29 ms, 95% CI [−262.30, −30.22], *p*__holm_ = 0.003, *d* = −0.87; AVGPs–NCSs: ΔM = −176.34 ms, 95% CI [−291.11, −61.56], *p*__holm_ = 3.962 × 10^−4^, *d* = −1.05), whereas TTPs did not differ from AVGPs (*p*__holm_ = 0.991) and AGAs did not differ from NCSs (*p*__holm_ = 0.991). The distribution of presentation duration thresholds across groups in Subtests 2 and 3, stratified by eccentricity, is shown in Figure 6.

## 4. Discussion

The present study used an adapted UFOV paradigm to compare performance patterns across multiple visual attention components in four groups with different experience backgrounds. The findings showed that experience-related differences did not emerge uniformly across all Subtests, but became progressively clearer as demands on central–peripheral coordination and distractor control increased. The groups performed similarly under the low-control central identification condition. Under central–peripheral coordination demands, all experienced groups outperformed the untrained controls. When strong distractor competition was introduced, only table tennis players and action video game players maintained the advantage. Overall, this pattern supports a component-specific rather than a component-general account of experience-related visual attention advantages. It also suggests that the advantage pattern shown by open-skill athletes is more closely related to high visual attention demand experience than to the broader effects of long-term physical training.

### 4.1. Subtest 1: No Group Difference Under Low-Control Central Identification

No reliable group differences were observed in Subtest 1. In the classical UFOV framework, Subtest 1 is commonly regarded as an index of central processing speed (Wood & Owsley, 2014). However, given the task format used in the present study, participants did not complete a pure speeded-response task, but rather a relatively simple central identification task. We therefore interpret this condition more cautiously as reflecting basic central processing efficiency and central identification performance under relatively low attentional control demands. Under this condition, participants only needed to identify a central target rapidly, without allocating attention between central and peripheral locations or suppressing irrelevant stimuli under strong visual competition.

This pattern is broadly consistent with previous research. In action video game players, for example, superior performance in complex visual–attentional contexts does not necessarily extend to simple perceptual or low-control tasks (Mack et al., 2016; Van Ravenzwaaij et al., 2014). Similar patterns have also been reported in the sport domain: athletes in open-skill sports do not invariably outperform non-athletes in certain low-complexity visual attention tasks (Su et al., 2024) and athletes in closed-skill sports do not necessarily show stable advantages in basic visual discrimination or simple attention conditions (Russo & Ottoboni, 2019). Taken together, the present Subtest 1 results suggest that no reliable group difference was observed in this relatively simple central identification condition. Accordingly, this condition does not provide evidence for an overall advantage shared across the experienced groups.

### 4.2. Subtest 2: Experience-Related Differences Emerge Under Central–Peripheral Coordination Demands

In Subtest 2, all three experienced groups outperformed NCSs, whereas no significant differences were found among TTPs, AVGPs, and AGAs. In light of the task demands, this pattern suggests that experience-related differences became more apparent once the task extended beyond the relatively low-demand central identification required in Subtest 1 and further required participants to allocate attention effectively to a peripheral location while maintaining central identification. Consistent with the hypotheses outlined in the Introduction, Subtest 2 involved more goal-directed attentional allocation between central and peripheral regions. Accordingly, group differences were no longer as limited as in Subtest 1. The advantage shown by the experienced groups is also in line with previous findings. Both action video game experience and sustained physical training have been associated with moderate benefits in attentional resource allocation. Action video games continuously challenge divided attention and rapid spatial reallocation, and have been proposed to train visuospatial attentional allocation mechanisms (Dale & Green, 2015), thereby supporting better performance in tasks requiring the coordination of central identification and peripheral localization (Hubert-Wallander et al., 2011). Previous research has also suggested that table tennis training involving complex visual stimulation is associated with plastic changes in white matter structure, and that such changes are related to better performance in UFOV Subtest 2 (Zheng et al., 2024). In addition, a meta-analytic review focusing on children and adolescents reported that open-skill sports training, such as football, was associated with significant improvements in attention-related outcomes (Mao et al., 2024).

However, this finding does not uniquely support any single explanation. In particular, it cannot be simply attributed to experience in highly visually competitive environments. Notably, AGAs were assumed to have less exposure than TTPs and AVGPs to this type of intense visual competition, yet they still showed a comparable advantage in Subtest 2. In terms of task demands, although Subtest 2 increased the load on central–peripheral attentional coordination, it did not yet introduce strong distractor competition. It may therefore reflect a relatively broad level of central–peripheral coordination, spatial orientation, and task preparation efficiency, rather than mechanisms specific enough to distinguish among different experience backgrounds (Plummer et al., 2022). For AGAs, although aerobic gymnastics does not emphasize the continuous processing of rapidly changing external competitive stimuli in the same way as table tennis or action video games, long-term training in this domain may still involve relatively high demands on spatial orientation, action organization, perception–action coordination, and efficient task preparation and execution. These characteristics may be sufficient to support better performance under this condition (Bisagno et al., 2022; Hendrianto & Firmansyah, 2023). This interpretation is also consistent with previous sport research showing that highly trained athletes often demonstrate advantages in visuospatial attention and attentional resource allocation (Chen et al., 2023; Chueh et al., 2025; Moreau, 2021). Taken together, the relatively good performance of AGAs and AVGPs, as well as TTPs, in Subtest 2 may suggest that, in a central–peripheral coordination task that does not yet impose strong distractor competition, different forms of long-term experience may be associated with similar behavioral benefits through different pathways. In addition, given the cross-sectional design, this finding should be interpreted with caution. The observed group differences may be related to long-term experience, but they may also partly reflect pre-existing individual differences.

### 4.3. Subtest 3: Experience-Specific Advantages Emerge Under Stronger Distractor Competition

Once strong distractor competition was introduced, the distinction between a general coordination benefit and experience-specific control became clearer. In Subtest 3, the peripheral target was embedded in a dense distractor array, which substantially increased the demands on selective attention and the suppression of irrelevant visual information (Wood et al., 2012). Under this condition, performance no longer depended only on attentional coordination across visual space. It also depended on the ability to maintain task goals in the presence of competing stimuli and to use stronger top–down control to prioritize task-relevant information while suppressing irrelevant input. This pattern is consistent with our expectations. Experience-related advantages do not generalize uniformly across all attentional components. When successful performance depends on maintaining goals, prioritizing task-relevant information, and resolving competition between stimuli, individual differences are more likely to emerge. Only table tennis players and action video game players maintained a performance advantage under this condition. Both groups are regularly exposed to environments with rapid dynamics, multiple competing visual elements, and continuous demands to select task-relevant information. Their experience therefore appears to be more closely aligned with the selective target processing and sustained suppression of irrelevant visual information required in Subtest 3. As previous studies have shown, individuals with extensive experience in complex visual gaming environments exhibit enhanced selective attention (Green & Bavelier, 2003). They also show stronger distractor suppression and target selection in tasks involving complex visual stimulation, which has been interpreted as reflecting more efficient attentional reallocation mechanisms (Mishra et al., 2011). Table tennis players appear to show similar attentional characteristics. Converging evidence suggests that, under conditions of visual conflict, table tennis athletes demonstrate better selective attention and inhibitory control. This has been reflected in faster responses and more efficient neural indices in color–word Stroop and spatial Stroop tasks (Huang et al., 2024), stronger executive control within attentional networks (Wang et al., 2016), and more efficient response inhibition under both conscious and unconscious conditions (You et al., 2018). Additional evidence further suggests that elite table tennis players are more likely to adopt an externally focused and narrowly regulated attentional state. This may help them prioritize task-relevant information in the presence of competing stimuli (Noor et al., 2025). They also appear to show broader advantages in higher-level executive function and selective attention (Elferink-Gemser et al., 2018).

By contrast, although aerobic gymnastics also involves high physical demands and long-term training, its task context is typically more self-paced and places relatively lower demands on sustained visual competition and distractor filtering. It may therefore provide less experience related to sustained visual competition and distractor filtering (Bisagno et al., 2022; Silvestri et al., 2025). This pattern also suggests that the shared advantage observed under lower or moderate attentional demands does not automatically increase as task difficulty rises. More importantly, whether an advantage is maintained under higher-demand conditions appears to depend more on whether the control processes required by the task match the processing demands repeatedly engaged by prior experience. Accordingly, the advantage shown by table tennis players under conditions of greater visual attentional control demand appears to be more closely related to experience in dynamic and highly competitive visual environments, rather than to an explanation based solely on the broader benefits of long-term sports training.

### 4.4. Eccentricity: Increased Peripheral Difficulty Without Altering the Group Pattern

By manipulating peripheral eccentricity at 10° and 16°, the present study examined differences between near- and far-peripheral processing within the same measurement framework. Performance was significantly worse at 16° than at 10°, which is consistent with previous findings showing that peripheral localization and functional field processing become more difficult as eccentricity increases (Staugaard et al., 2016). This suggests that the eccentricity manipulation in the present study effectively increased peripheral processing demands.

More importantly, eccentricity did not interact significantly with group or Subtest, and the three-way interaction was also not significant. This suggests that the additional difficulty imposed by the farther peripheral condition was relatively similar across groups and did not substantially alter the relative performance pattern observed in Subtests 2 and 3. This null interaction may suggest that the observed experience-related differences were more related to attentional control under interference than to increased peripheral spatial demand per se. In other words, increasing eccentricity raised overall task difficulty, but did not alter the main pattern observed in the present study, namely that experience-related differences varied across attentional components.

## 5. Limitations

Several limitations should be acknowledged. First, the present study used a cross-sectional design and therefore does not allow causal inference regarding the relationship between long-term experience and visual attention advantages. The observed findings may reflect differences associated with experience background, but may also have been partly influenced by pre-existing individual differences and related selection effects. Second, although verbal screening was used during recruitment to reduce overlap in experience backgrounds across groups, this procedure was not based on standardized questionnaires or more fine-grained quantitative assessments. Residual influences of prior visual- or sport-related experience therefore cannot be fully ruled out, particularly in the control group. Third, the present study relied only on behavioral indices to assess performance on the adapted UFOV task, and Subtest 2 and Subtest 3 were administered in a fixed order. Although short practice phases and rest breaks were included to reduce immediate carryover and fatigue, potential order-related influences, including forward carryover from Subtest 2 to Subtest 3, cannot be completely ruled out. As a result, the current data cannot reveal more fine-grained processing mechanisms, and potential order effects, such as practice, strategy adjustment, or fatigue, cannot be completely excluded. Future research could combine eye-tracking or neurophysiological measures with more refined task arrangements to further test the robustness of the present findings.

## 6. Conclusions

The findings indicate that experience-related advantages in visual attention are component-specific rather than global. No group differences emerged under low-control central identification, whereas differences became evident when performance relied on central–peripheral coordination and were most clearly separated under high visual competition.

## Figures and Tables

**Table 1:** Demographic characteristics.

Characteristic	TTPs (*n* = 24)	AVGPs (*n* = 25)	AGAs (*n* = 28)	NCSs (*n* = 27)	F/χ^2^/t	*p*
Age (years)	19.04 ± 1.20	20.00 ± 1.71	20.18 ± 1.61	19.93 ± 1.77	2.52	0.063
Gender (M/F)	13/11	7/18	14/14	14/13	4.46	0.216
Training years	10.63 ± 2.68	/	11.14 ± 3.93	/	−0.55	0.588
weekly training or gaming hours	16.71 ± 12.25	14.56 ± 6.03	18.54 ± 6.92	/	1.38	0.259

## Data Availability

The datasets generated and analyzed during this study include de-identified baseline information (e.g., age, years of training and weekly training hours), UFOV performance data from the three Subtests, and statistical outputs. Data sharing complies with institutional ethics guidelines and is available from the corresponding author upon reasonable request.

## Abbreviations

The following abbreviations are used in this manuscript:

TTPs	Table tennis players
AVGPs	Action video game players
AGAs	Aerobic gymnastics athletes
NCSs	Non-trained college students
